# A dataset of digital holograms of normal and thalassemic cells

**DOI:** 10.1038/s41597-023-02818-4

**Published:** 2024-01-02

**Authors:** Vahid Abbasian, Arash Darafsheh

**Affiliations:** 1 Department of Radiation Oncology, Washington University School of Medicine in St. Louis, St. Louis, MO 63110 USA; 2 Imaging Science Program, McKelvey School of Engineering, Washington University in St. Louis, St. Louis, MO 63130 USA; 3 Department of Physics, Institute for Advanced Studies in Basic Sciences (IASBS), Zanjan, 45137-66731 Iran

**Keywords:** 3-D reconstruction, Interference microscopy

## Abstract

Digital holographic microscopy (DHM) is an intriguing medical diagnostic tool due to its label-free and quantitative nature, providing high-contrast images of phase samples. By capturing both intensity and phase information, DHM enables the numerical reconstruction of quantitative phase images. However, the lateral resolution is limited by the diffraction limit, which prompted the recent suggestion of microsphere-assisted DHM to enhance the DHM resolution straightforwardly. The use of such a technique as a medical diagnostic tool requires testing and validation of the proposed assays to prove their feasibility and viability. This paper publishes 760 and 609 microsphere-assisted DHM images of normal and thalassemic red blood cells obtained from a normal and thalassemic male individual, respectively.

## Background & Summary

Optical microscopy is the most widely used technique for pathological inspection of biological samples^1^. Conventional microscopy provides a two-dimensional (2D) image corresponding to the light intensity distribution across the specimen. Digital holographic microscopy (DHM), however, provides a phase image thanks to its ability to capture both intensity and phase information across the specimen^2^. DHM is an interesting technique to obtain phase images of cells for automated disease identification^3^. In recent advancements, DHM, combined with hierarchical machine learning, has been successfully applied for the differential diagnosis of hereditary anemias, demonstrating the technique’s capability to classify healthy and pathological red blood cells (RBCs) from phase-contrast maps with high accuracy^4^. However, like other optical microscopy methods, the lateral spatial resolution in DHM is subject to the diffraction limit^5^. There has been a significant amount of interest in developing multiple approaches to overcome this limitation toward optical super-resolution imaging^6^. This can be safely achieved by synthetically increasing the numerical aperture (NA) of the detection system, which manipulates the system’s degrees of freedom to synthetically increase the NA, thereby enhancing lateral resolution^7,8^. In this amongst, microsphere-assisted microscopy (MAM) stands out for its simplicity, cost-effectiveness, and the advantage of enhancing resolution in all lateral directions simultaneously, making it particularly suitable for real-time imaging of dynamic live specimens^6,9,10^. In MAM, a transparent micron-scale sphere is positioned over the specimen to enhance lateral resolution compared to the same system operating in the absence of the microsphere^11^. MAM, thanks to its broadband and versatile nature, can be incorporated into various microscopy techniques, including confocal^12^,fluorescent^13,14^, two-photon and second-harmonic generation microscopies^15^, profilometry^16,17^, polarimetry^18^ and DHM^19–21^.

A normal RBC typically has a biconcave disc shape, resembling a shallow donut or a disk with a depressed center. Its diameter ranges from 6 to 8 μm while reaching a thickness of approximately 2 μm at the edges. This specific geometry provides a high surface area-to-volume ratio, facilitating the exchange of oxygen and carbon dioxide. These morphological characteristics, in conjunction with the absence of a nucleus, contribute to the RBC’s ability to navigate through narrow capillaries and ensure effective oxygen delivery to tissues throughout the body. However, certain diseases or health conditions can lead to alterations in RBC morphology. For example, in thalassemia minor, changes in the RBC membrane composition lead to increased rigidity and a propensity for deformation. Thalassemia encompasses various forms, including thalassemia major, which is more severe, and thalassemia minor, which occurs when an individual inherits a faulty gene from only one parent^22,23^. These inherited blood disorders can be detected and diagnosed through medical laboratory tests such as a complete blood count (CBC), which can identify anemia, and a hemoglobin electrophoresis test, which can detect abnormal forms of hemoglobin in the RBCs. These diagnostic tests play a crucial role in accurately identifying and classifying different types of thalassemia, allowing healthcare professionals to provide appropriate management and treatment strategies. The morphological study of thalassemic RBCs reveals distinct characteristics associated with this genetic condition. When examining the RBCs of individuals with thalassemia minor, several morphological changes are typically observed. These changes include microcytosis, where the cells appear smaller than normal; hypochromia, resulting in paler cells due to decreased hemoglobin content; the presence of target cells, also known as codocytes, which display a bullseye-like appearance; anisocytosis, indicating variations in cell size; and poikilocytosis, representing a variety of abnormal cell shapes. The severity and presence of these morphological changes can vary among individuals, but microcytosis and hypochromia are the most common types observed. Understanding these distinct morphological features of RBCs in thalassemia minor is crucial for diagnosing and differentiating this condition from other forms of anemia. Therefore, DHM is expected to accurately distinguish thalassemic RBCs from normal ones, as it can provide precise quantitative morphometric information about phase objects.

Recorded holograms of cells offer a wealth of information beyond just visualizing their structure. These holograms enable the extraction of various features, such as cell membrane roughness, thickness distribution behavior, cell circularity, volumetric aspect ratio, and cell convexity^24–26^. Evaluating these parameters provides crucial insights into cell health, integrity, deformations, and response to external stimuli. For example, assessing membrane roughness allows researchers to study cell-membrane interactions and identify pathological conditions. Analyzing thickness distribution helps understand variations in cellular deformations, while circularity indicates deviations in cellular function. Volumetric aspect ratio aids in differentiating cell types and detecting changes caused by diseases or environmental factors. Additionally, measuring cell convexity reveals alterations in adhesion, cytoskeletal rearrangements, and membrane protrusions^27^. These derived parameters serve as identification markers and are vital for investigating the impact of organic compounds on cell structures, enabling advancements in fields like medicine, biology, and materials science^3,28,29^. Furthermore, DHM offers the unique advantage of measuring the dry mass distribution of RBCs, which correlates with hemoglobin content and, consequently, the oxygen transport capacity of the blood—a feature that aligns with conventional mean corpuscular hemoglobin (MCH) and mean corpuscular volume (MCV) measures used in blood screening^30^. This capability is particularly significant in the context of RBCs, as variations in hemoglobin concentration can lead to changes in optical thickness and cell membrane properties, impacting the mechanical and physical characteristics of the cells^30^.

It has been suggested that MAM combined with DHM has the potential for cell identification^21,31,32^. when combined, MAM enhances the lateral resolution, while DHM provides high axial resolution to create a phase map. To prove the principle for a medical diagnostic application, we already tested microsphere-assisted DHM to differentiate between a small number of thalassemia minor RBCs (tRBCs) and normal RBCs (nRBCs)^31^. However, only a volumetric analysis of such cells was performed, and it was limited to 140 RBCs from either case. Recorded digital holograms could be used to perform a more rigorous morphologic study to accurately determine the sub-type of the tRBCs. Developing such a technique requires training on a large data set^33–35^. Due to the novelty of microsphere-assisted DHM, here for the first time, we share DHM data of 760 images of nRBCs and 609 images of tRBCs.

## Methods

### Principles of DHM

Holography involves a two-step process, beginning with the recording of an interference pattern between a reference beam and an object beam, followed by the reconstruction of a phase map through the illumination of the recorded hologram with coherent light. During recording, a coherent light beam is split into two arms, with one arm directed towards the object, causing light to scatter onto the image plane, while the other arm reaches the image plane intact^36,37^. The beams’ superposition on the image plane creates a hologram, encoding the intensity and phase information required for faithful object reconstruction. Conventional holography involves capturing holograms on photographic plates, which then undergo development and fixation processes similar to traditional photography.

The interaction of light waves with objects leads to alterations in both their amplitude and phase, which can be attributed to the unique physical and structural characteristics of the objects. This can be expressed by the spatial part of the reflected or transmitted light wave as follows:

1 $$\vec{E}(x,y)={\vec{E}}_{0}(x,y){e}^{i\phi (x,y)},$$

where ${E}_{0}(x,y)$ and $\phi (x,y)$ represent the amplitude and phase of the light wave, respectively. The amplitude changes are influenced by light absorption in the object, while the phase changes are related to variations in thickness (for transmissive objects) or surface height profile (for reflective objects). However, conventional image detectors can only capture the intensity of the light wave $(I(x,y)={|{\vec{E}}_{0}(x,y)|}^{2})$, resulting in the loss of phase information, which carries valuable phase object details. To preserve and detect phase information, holography relies on superposing the object’s light wave with a reference light wave before reaching the detector. The interference pattern of the object (sample) beam, ${\vec{E}}_{0{\rm{s}}}(x,y){e}^{i{\phi }_{{\rm{s}}}(x,y)}$, and the reference beam, ${\vec{E}}_{0{\rm{r}}}(x,y){e}^{i{\phi }_{{\rm{r}}}(x,y)}$, is given by:

2 $$I(x,y)={|{\vec{E}}_{0{\rm{s}}}(x,y)|}^{2}+{|{\vec{E}}_{0{\rm{r}}}(x,y)|}^{2}+2\,[{\vec{E}}_{0{\rm{s}}}(x,y).{\vec{E}}_{0{\rm{r}}}(x,y)]\,\cos [{\phi }_{{\rm{s}}}(x,y)-{\phi }_{{\rm{r}}}(x,y)].$$

where *E*_0s_ and *ϕ*_s_ represent the amplitude and phase of the sample beam, and *E*_0r_ and *ϕ*_r_ represent the amplitude and phase of the reference beam, respectively.

The phase difference, ${\phi }_{{\rm{s}}}-{\phi }_{{\rm{r}}}$, is directly related to the difference in optical path length between the two light waves, which allows for the measurement of the reflective object’s height or the transmissive object’s thickness at any given point (*x*, *y*). The last two terms in the equation contain comprehensive information about the object wave. During the reconstruction process, these terms give rise to a virtual image and a real image. However, the first two terms in the equation, known as the zero-order terms, introduce noise into the final reconstructed images and must be eliminated as part of the reconstruction process^38^.

Reconstruction of the holograms is achieved by illuminating them with a reference laser beam. However, conventional holography has limitations such as the need for photographic development, additional reconstruction facilities, and difficulties in studying dynamic samples. Digital holography overcomes these drawbacks by recording holograms directly onto a digital camera, eliminating the need for development processes^39^. Numerical reconstruction is performed on a computer, simulating the illumination and diffraction of the holograms based on scalar diffraction theory. One notable application of digital holography is DHM, which integrates with conventional microscopes to provide high-resolution, quantitative information about the phase structure of microscopic samples^39,40^.

### Sample preparation

Blood smears were obtained with written informed consent, following approval by the Institutional Review Board of the Institute for Advanced Studies in Basic Sciences, from a healthy subject and a subject who had been tested positive for thalassemia minor. Following proper sterilization techniques, a finger-pricking device was used to create a small puncture on the side of the finger. A small drop of the blood sample was carefully collected each time and placed near the edge of a clean glass slide. Another slide was positioned at an angle of less than 45 degrees against the first slide, allowing the blood to naturally spread along the edge. The second slide was gently pushed forward along the first slide, ensuring a uniform and thin smear. To prevent clotting and distortion of the cells, the smear was air-dried at room temperature for about 10 minutes, while avoiding exposure to heat sources and direct sunlight. Hence, thin blood smears were successfully created on two separate glass slides red each time, and subsequently used for recording the holograms of nRBCs and tRBCs. Hence, each hologram is an exclusive representation of a single RBC, ensuring the uniqueness of our dataset.

### Experimental setup

**Fig. 1:**
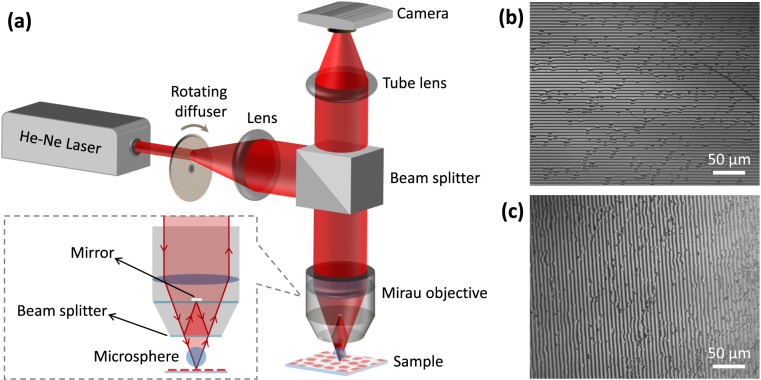
(**a**) Scheme of the used DHM setup for collecting digital holographic data from normal and thalassemic RBCs. (**b**) and (**c**) recorded full-field holograms of nRBC and tRBC, respectively, without using a microsphere in the setup.

Our DHM setup is shown in Fig. 1a. A 2 mW He-Ne laser providing coherent light at 632.8 nm wavelength was used as the light source. Passing the light through a rotating diffuser helped to reduce the speckle noise. The beam was collimated through a converging lens with a 10 cm focal distance and sent to a Mirau interferometric objective (Nikon, NA = 0.3, 10×) through a cube beam splitter. Mirau objectives offer a compact and highly temporally stable geometry in reflection mode for high-resolution phase imaging. In order to elevate the lateral resolution, a silica microsphere with 234 μm diameter and 1.46 index of refraction was placed within the working distance of the objective lens. This was achieved by attaching the microsphere with an optical adhesive to the end of an optical fiber, which served as a lever. The fiber was then carefully inserted through a micropipette tip to ensure stability. Subsequently, an x-y-z micropositioner was connected to the micropipette tip, enabling precise manipulation and positioning of the microsphere over the specimen. The possibility to vary the microsphere’s size and position provides a large range of resolutions, magnification factors, and field of view (FoV), allowing one to opt for a desired imaging property^31,41^. In MAM, it is critical that the feature of interest placed within the FoV of the microsphere. Several techniques such as self-assembly and micro-manipulation have been proposed for microsphere positioning^6^. Connecting the sphere to the optical fiber allowed for convenient adjustment of the sphere’s position in our setup. In bright-field MAM, image aberrations such as pincushion distortion have been reported^42^. In DHM, however, imaging aberrations due to the microsphere can be minimized through a reference hologram as suggested in the literature^43^.

The built-in plate beam splitter at the front aperture of the objective divided the incoming beam into two parts (see the inset of Fig. 1a). One part was reflected back onto a small mirror within the objective, serving as the reference beam. The other part was transmitted through the beam splitter, passed through the microsphere, and then focused onto the sample. Upon reflection from the sample, the diffracted wave containing high spatial frequencies was collected by the microsphere. The interfering beams traveled along a common path and created an interference pattern at the sensor plane, which was achieved using a tube lens with a focal length of 16 cm. For recording the pattern as digital holograms, we utilized a digital camera (DCC1545M, Thorlabs, 8-bit dynamic range, 5.2 μm pixel pitch). The digital holograms were saved as 8-bit images with a resolution of 1280 pixels by 1024 pixels in the BMP format. Figure 1b,c show the full-frame holograms of the nRBCs and tRBCs, respectively, recorded prior to the insertion of a microsphere in the presented setup primarily aiming at calibrating the system. The change in the direction of the interference fringes is due to an inadvertent rotation of the camera.

Quantification of the achieved lateral resolution gain by the microsphere is beyond the scope of this work. In principle, standard resolution tests such as USAF targets can be used as in conventional microscopy^6^. However, the proof-of-concept experiment was already performed on a resolution test target and a standard grating, and the various adjustments and effective parameters on the imaging performance were investigated^31^. The presented MS-assisted setup successfully revealed the structure of the grating with a period of 732 nm, which is beyond the resolution limit of a 10× Mirau objective with NA = 0.3 and theoretically needs a minimum effective NA of 0.86 (NA = *λ*/*d*, where *λ* = 632.8 nm is the wavelength of light and *d* is the period of the grating). This is achieved by our rather large MS for the present experiment, yet the setup has the potential to offer a wide range of improved resolution and magnification factors depending on the physical properties of the MS and its vertical position between the sample and objective^31^. However, pursuing higher resolution and magnification levels may come at the cost of a limited FoV for the sample under study, which is a trade-off similar to that observed in MAM^6,44^. Nanosphere-based approaches have also been mentioned to enhance the resolution and FoV^45^; however, their integration in DHM setups has not been investigated.

### Numerical reconstruction process

Various numerical methods have been proposed and developed for the reconstruction of the recorded digital holograms^39,46^. We used the angular spectrum propagation approach (ASP)^40,47^, which is elucidated as follows. The reconstruction process involves obtaining the complex amplitude, encompassing both amplitude and phase information. Specifically, the light wave at the hologram’s plane denoted as *E*_s_(*x*, *y*, *z* = 0), is computed by numerically illuminating the recorded digital hologram with the reference wave *E*_r_(*x*, *y*). The angular spectrum of *E*_s_(*x*, *y*, 0), corresponding to its Fourier transform, can be expressed as:

3 $${\mathscr{F}}\{{E}_{{\rm{s}}}(x,y)\}={\mathop{E}\limits^{ \sim }}_{{\rm{s}}}(\xi ,\eta )=\int {\int }_{-\infty }^{\infty }{E}_{{\rm{s}}}(x,y,0){e}^{-2\pi i(\xi x+\eta y)}dxdy.$$

The variables *ξ* and *η* represent the spatial frequencies in the *x* and *y* directions, respectively. Within the Fourier domain, the spatial frequencies corresponding to the undiffracted beam and the virtual image undergo a filtering process. Subsequently, the modified light wave is obtained by performing an inverse Fourier transform, yielding the following result:

4 $${E}_{{\rm{s}}}^{{\rm{F}}}(x,y,0)={{\mathscr{F}}}^{-1}\{{\mathop{E}\limits^{ \sim }}_{{\rm{s}}}^{{\rm{F}}}(\xi ,\eta ,0)\}=\int {\int }_{-\infty }^{\infty }{\mathop{E}\limits^{ \sim }}_{{\rm{s}}}^{{\rm{F}}}(\xi ,\eta ,0){e}^{2\pi i(\xi x+\eta y)}d\xi d\eta ,$$

where ${\widetilde{E}}_{{\rm{s}}}^{{\rm{F}}}$ represents the filtered angular spectrum of *E*_s_. The complex amplitude at *z* = *d* is obtained by free-space propagation of ${E}_{{\rm{s}}}^{{\rm{F}}}(x,y,0)$ over a distance *d*:

5 $${E}_{{\rm{s}}}^{{\rm{F}}}(x,y,d)=\int {\int }_{-\infty }^{\infty }{\widetilde{E}}_{{\rm{s}}}^{{\rm{F}}}(\xi ,\eta ,0){e}^{ikd\sqrt{1-{\lambda }^{2}{\xi }^{2}-{\lambda }^{2}{\eta }^{2}}}{e}^{2\pi i(\xi x+\eta y)}d\xi d\eta .$$

where *λ* represents the wavelength of the light source. The entire process can be summarized by the following equation:

6 $${E}_{{\rm{s}}}^{{\rm{F}}}(x,y,d)={{\mathscr{F}}}^{-1}\{{[{\mathscr{F}}\{{E}_{{\rm{s}}}(x,y,0)\}]}^{{\rm{F}}}{e}^{ikd\sqrt{1-{\lambda }^{2}{\xi }^{2}-{\lambda }^{2}{\eta }^{2}}}\}.$$

The intensity of the object *I*_s_, and the phase of the object *ϕ*_s_, can be calculated from the complex amplitude as follows:

7 $${I}_{{\rm{s}}}(x,y,z)={\left|{E}_{{\rm{s}}}^{{\rm{F}}}(x,y,z)\right|}^{2},$$

8 $${\phi }_{{\rm{s}}}(x,y,z)={\tan }^{-1}\frac{{\rm{\Im }}[{E}_{{\rm{s}}}^{{\rm{F}}}(x,y,z)]}{{\rm{\Re }}[{E}_{{\rm{s}}}^{{\rm{F}}}(x,y,z)]}.$$

Equation (7) produces microscopy-like intensity images. However, DHM goes a step further by enabling numerical refocusing at specific axial planes, eliminating the need for mechanical adjustments in traditional microscopy. This non-invasive refocusing feature offers flexible and precise exploration of the object’s phase structure without physically moving the microscope components. The phase map of the object is obtained through Eq. (8) by calculating the arctangent of the imaginary and real components of the complex amplitude ${E}_{{\rm{s}}}^{{\rm{F}}}(x,y,z)$. However, this calculation results in a phase map with discontinuities due to its limited range of $[-\pi /2,\pi /2]$. To address this issue, a phase unwrapping process is required to remove the phase ambiguities caused by the discontinuities. To this end, we used Goldstein’s branch-cut unwrapping algorithms^48^. By unwrapping the phase maps, continuous and accurate phase maps of the samples can be obtained as:

9 $${\phi }_{{\rm{s}}}(x,y)=\frac{2\pi }{\lambda }n{T}_{{\rm{s}}}(x,y),$$

where *n* is the refractive index of the medium, and *T*_s_(*x*, *y*) is the thickness distribution of the sample.

## Data Records

The data record has been deposited at figshare repository^49^. Digital holograms in BMP format have been stored in two folders named “nRBC” and “tRBC” corresponding to the normal and thalassemic cases, respectively.

## Technical Validation

**Fig. 2:**
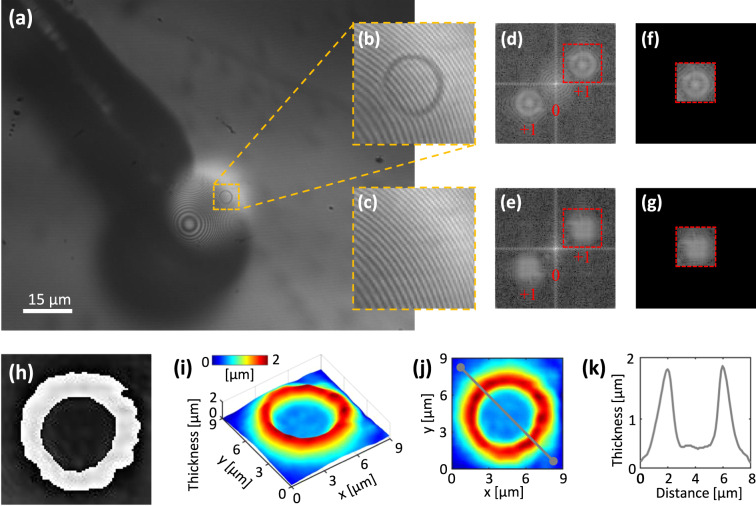
(**a**) Recorded full-field hologram of an nRBC by microsphere-assisted DHM using a 234 μm diameter microsphere. The fiber lever connected to the microsphere can be seen in a panel with a dark grey appearance. (**b**) expanded view of the region of interest around the RBC enclosed by the dashed square depicted in panel (**a**), and (**c**) its associated reference hologram. (d) and (e) Fourier spectra of the object (**b**) and reference (**c**) holograms, respectively, which include three frequency regions associated with virtual and real images, as well as the zero-order term of Eq. (2). Either of the regions can be selected, shifted into the center of the domain, and subjected to proper numerical propagation to reach the complex amplitude of the image. (**f**) and (**g**) associated filtered frequency spectra after shifting and centering. To select the desired frequency component, a binary mask was applied, and then were then propagated to the best focus plane located at a distance *z* = *d* from the hologram plane according to Eq. (5). The phase map of the sample can be derived from the complex amplitude as discussed in Eq. (8), leading to the phase contrast image of the nRBC shown in panel (**h**). This image was obtained by subtracting the computed reference phase distribution from the object hologram to remove any image distortions. (**i**) Computed thickness distribution, and (**j**) its 2D view, using Eq. (9) after unwrapping the phase image depicted in (**h**). (**k**) Cross-sectional profile along the arbitrary line indicated in panel (**j**).

**Fig. 3:**
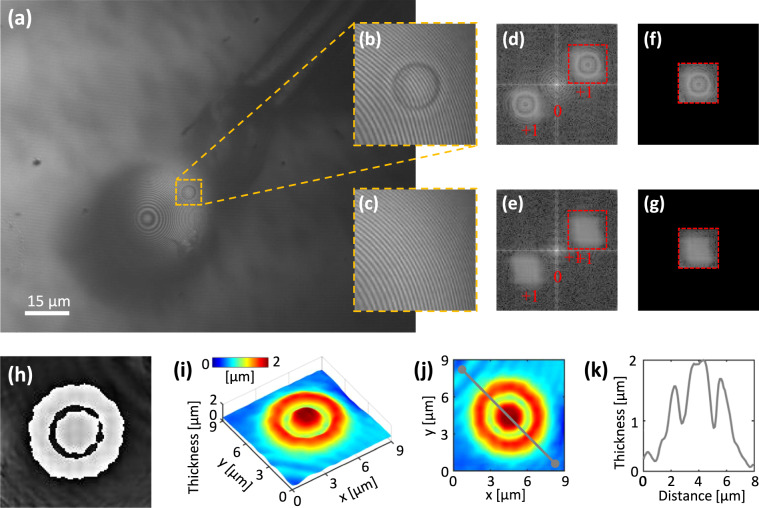
(**a**) Recorded full-field hologram of a tRBC by the presented DHM setup using the 234 μm diameter microsphere. (**b**) expanded view of the region of interest around the tRBC enclosed by the dashed square (a), and (**c**) its associated reference hologram. (**i**) reconstructed phase map of the RBC, and (**j**) its 2D view. (**k**) Cross-sectional profile along the arbitrary line indicated in panel (**i**). Refer to the caption of Fig. 2 for more details.

Typical microsphere-assisted DHM images of an nRBC and tRBC along with the corresponding reconstruction procedures are summarized in Figs. 2, 3, respectively. Figures 2a, 3a show the recorded full-field holograms after incorporating a 234 μm diameter microsphere into the DHM setup. The unfocused images of the microsphere attached to the fiber optics can be clearly seen in Figs. 2a, 3a. It is important to note that the concentric rings surrounding the center of the microsphere are Newton ring artifacts and should not be mistaken for the RBCs. Figures 2b, 3b provide an enlarged view of the region of interest (RoI) inside the rectangles shown in Figs. 2a, 3a,respectively, where the parallel interference fringes are prominently visible. These RoI digital holograms underwent numerical reconstruction using the previously described ASP approach. In order to compensate for any potential contamination and aberrations of the optical train from the extracted data, we considered reference conjugated hologram trick^43^. This was achieved by considering a cell-free region of the recorded holograms as a reference hologram. the associated reference holograms of the RoI object holograms are shown in Figs. 2c, 3c. The object and reference holograms of each RBC were reconstructed individually, and by subtracting the background hologram phase from them, the phase difference distribution *ϕ*_RBC_(*x*, *y*) was obtained, following Eq. 8. This subtraction process effectively cancels out the phase change introduced by the optical elements, which remained constant throughout the exposures. Panels (h) in Figs. 2, 3 show the phase contrast images obtained through the phase subtraction, respectively. The associated reconstructed thickness distribution computed through the Eq. 9 for the two types of RBCs are shown in panels (i-k) in Figs. 2, 3.

## Usage Notes

The whole data can be downloaded from figshare repository. The data can be used with a wide range of image processing software and packages such as MATLAB and Python among others.

## Data Availability

The technical details of the reconstruction algorithm used to generate phase images are described in detail in the Methods section, providing the necessary information for reproducibility of the phase reconstruction of the presented dataset.

## References

[1] Morris JD, Payne CK (2019). Microscopy and cell biology: new methods and new questions. Annual review of physical chemistry.

[2] Tahara T, Quan X, Otani R, Takaki Y, Matoba O (2018). Digital holography and its multidimensional imaging applications: a review. Microscopy.

[3] Anand A, Moon I, Javidi B (2017). Automated disease identification with 3-d optical imaging: a medical diagnostic tool. Proceedings of the IEEE.

[4] Memmolo P (2022). Differential diagnosis of hereditary anemias from a fraction of blood drop by digital holography and hierarchical machine learning. Biosensors and Bioelectronics.

[5] Darafsheh A, Abbasian V (2023). Dielectric microspheres enhance microscopy resolution mainly due to increasing the effective numerical aperture. Light: Science & Applications.

[6] Darafsheh A (2022). Microsphere-assisted microscopy. Journal of Applied Physics.

[7] Mico V, Zalevsky Z, Garca-Martnez P, Garca J (2006). Synthetic aperture superresolution with multiple off-axis holograms. JOSA A.

[8] Paturzo M, Ferraro P (2009). Correct self-assembling of spatial frequencies in super-resolution synthetic aperture digital holography. Optics letters.

[9] Luk’yanchuk BS, Paniagua-Domnguez R, Minin I, Minin O, Wang Z (2017). Refractive index less than two: photonic nanojets yesterday, today and tomorrow. Optical Materials Express.

[10] Trukhova A, Pavlova M, Sinitsyna O, Yaminsky I (2022). Microlens-assisted microscopy for biology and medicine. Journal of Biophotonics.

[11] Darafsheh, A. *Optical super-resolution and periodical focusing effects by dielectric microspheres*. Ph.D. thesis, The University of North Carolina at Charlotte (2013).

[12] Darafsheh, A., Limberopoulos, N. I., Derov, J. S., Walker, D. E. & Astratov, V. N. Advantages of microsphere-assisted super-resolution imaging technique over solid immersion lens and confocal microscopies. *Applied Physics Letters* **104** (2014).

[13] Darafsheh A, Guardiola C, Palovcak A, Finlay JC, Cárabe A (2015). Optical super-resolution imaging by high-index microspheres embedded in elastomers. Optics Letters.

[14] Yang H, Moullan N, Auwerx J, Gijs MA (2014). Super-resolution biological microscopy using virtual imaging by a microsphere nanoscope. Small.

[15] Tehrani KF, Darafsheh A, Phang S, Mortensen LJ (2018). Resolution enhancement of 2-photon microscopy using high-refractive index microspheres. Proceedings of SPIE.

[16] Kassamakov I (2017). 3d super-resolution optical profiling using microsphere enhanced mirau interferometry. Scientific reports.

[17] Pahl T, Hüser L, Hagemeier S, Lehmann P (2022). Fem-based modeling of microsphere-enhanced interferometry. Light: Advanced Manufacturing.

[18] Abbasian V, Moradi A-R (2020). Microsphere-assisted super-resolved mueller matrix microscopy. Optics Letters.

[19] Abbasian V, Rasouli S, Moradi A-R (2019). Microsphere-assisted self-referencing digital holographic microscopy in transmission mode. Journal of Optics.

[20] Abbasian V, Darafsheh A, Moradi A-R (2023). Simple high-resolution 3d microscopy by a dielectric microsphere: a proof of concept. Opt. Lett..

[21] O’Connor T, Anand A, Javidi B (2020). Field-portable microsphere-assisted high resolution digital holographic microscopy in compact and 3d-printed mach-zehnder interferometer. OSA Continuum.

[22] Keohane, E., Otto, C. N. & Walenga, J. *Rodak’s hematology-e-book: clinical principles and applications* (Elsevier Health Sciences, 2019).

[23] Hoffman, R. *et al*. *Hematology: basic principles and practice* (Elsevier Health Sciences, 2013).

[24] Jaferzadeh K, Moon I (2015). Quantitative investigation of red blood cell three-dimensional geometric and chemical changes in the storage lesion using digital holographic microscopy. Journal of biomedical optics.

[25] Moon I, Javidi B, Yi F, Boss D, Marquet P (2012). Automated statistical quantification of three-dimensional morphology and mean corpuscular hemoglobin of multiple red blood cells. Optics express.

[26] Yi F, Moon I, Javidi B (2016). Cell morphology-based classification of red blood cells using holographic imaging informatics. Biomedical optics express.

[27] Yi F, Lee C-G, Moon I-K (2012). Statistical analysis of 3d volume of red blood cells with different shapes via digital holographic microscopy. Journal of the Optical Society of Korea.

[28] Anand A, Chhaniwal V, Patel N, Javidi B (2012). Automatic identification of malaria-infected rbc with digital holographic microscopy using correlation algorithms. IEEE Photonics Journal.

[29] Abbasian V, Akhlaghi EA, Charsooghi MA, Bazzar M, Moradi A-R (2018). Digital holographic microscopy for 3d surface characterization of polymeric nanocomposites. Ultramicroscopy.

[30] Joglekar, M. *et al*. Imaging the effect of hemoglobin on properties of rbcs using common-path digital holographic microscope. In *European Conference on Biomedical Optics*, 104140W (Optica Publishing Group, 2017).

[31] Aakhte M (2017). Microsphere-assisted super-resolved mirau digital holographic microscopy for cell identification. Applied optics.

[32] Abbasian V (2018). Super-resolved microsphere-assisted mirau digital holography by oblique illumination. Journal of Optics.

[33] O’Connor T, Anand A, Andemariam B, Javidi B (2020). Deep learning-based cell identification and disease diagnosis using spatio-temporal cellular dynamics in compact digital holographic microscopy. Biomedical Optics Express.

[34] Yi F, Moon I, Javidi B (2017). Automated red blood cells extraction from holographic images using fully convolutional neural networks. Biomedical optics express.

[35] O’Connor T, Shen J-B, Liang BT, Javidi B (2021). Digital holographic deep learning of red blood cells for field-portable, rapid covid-19 screening. Optics Letters.

[36] Gabor D (1948). A new microscopic principle. Nature.

[37] Leith EN, Upatnieks J (1962). Reconstructed wavefronts and communication theory. JOSA.

[38] Hariharan, P. & Hariharan, P. *Optical Holography: Principles, techniques and applications* (Cambridge University Press, 1996).

[39] Kim, M. K. & Kim, M. K. *Digital holographic microscopy* (Springer, 2011).

[40] Anand A, Chhaniwal VK, Javidi B (2010). Real-time digital holographic microscopy for phase contrast 3d imaging of dynamic phenomena. Journal of display technology.

[41] Darafsheh A (2016). Comment on ‘super-resolution microscopy by movable thin-films with embedded microspheres: Resolution analysis’[ann. phys.(berlin) 527, 513 (2015)]. Annalen der Physik.

[42] Darafsheh, A., Walsh, G. F., Dal Negro, L. & Astratov, V. N. Optical super-resolution by high-index liquid-immersed microspheres. *Applied Physics Letters* **101** (2012).

[43] Colomb T (2006). Total aberrations compensation in digital holographic microscopy with a reference conjugated hologram. Optics express.

[44] Darafsheh A (2021). Photonic nanojets and their applications. Journal of Physics: Photonics.

[45] McLeod E (2014). Tunable vapor-condensed nanolenses. ACS nano.

[46] Osten W (2014). Recent advances in digital holography. Applied optics.

[47] Goodman, J. W. *Introduction to Fourier optics* (W. H. Freeman and Company, 2017).

[48] Gutmann B, Weber H (2000). Phase unwrapping with the branch-cut method: role of phase-field direction. Applied optics.

[49] Abbasian V, Darafsheh A (2023). figshare.

